# Case Report: Long-term improvement after acupotomy in advanced ankylosing spondylitis with sacroiliac joint fusion

**DOI:** 10.3389/fsurg.2025.1727275

**Published:** 2025-12-12

**Authors:** Yu Chen, Zixi Ye, Yangguang Yuan, Xiangrui Cui, Dongyue Guo, Liyong Zhang, Qiuxia Ding

**Affiliations:** 1 Respiratory and Critical Care Department, Shenzhen Hospital of Shanghai University of Traditional Chinese Medicine, Shenzhen, Guangdong, China; 2 Famous Traditional Chinese Medicine Clinic, Shenzhen Hospital of Shanghai University of Traditional Chinese Medicine, Shenzhen, Guangdong, China; 3 Luohu Clinical Institute, Shantou University Medical College, Shantou, Guangdong, China; 4 Department of Radiology, The Third Affiliated Hospital of Shenzhen University (Luohu Hospital Group), Shenzhen, China; 5 Medical Laboratory, Shenzhen Luohu People’s Hospital, Shenzhen, Guangdong, China; 6 Traditional Chinese Medicine (TCM) Diagnosis and Treatment Center, Changsha Fourth Hospital (Changsha Hospital of Integrated Traditional Chinese and Western Medicine), Changsha, Hunan, China

**Keywords:** ultrasound, thoracolumbar fascia, posture correction, minimally invasive surgery, dynamic release, trigger points

## Abstract

**Background:**

Evidence for acupotomy in managing advanced ankylosing spondylitis (AS) with extensive bony fusion, particularly in treatment-refractory cases, remains limited. This report details its application and outcomes in one such patient.

**Case presentation:**

A 27-year-old male with advanced, active AS and bilateral sacroiliac joint fusion presented with severe low back pain, kyphosis, and markedly restricted mobility. His condition was refractory to prior conservative therapies, including NSAIDs and traditional acupuncture. He subsequently underwent five sessions of anatomy-guided acupotomy, targeting fascial adhesions in the thoracolumbar fascia, sacroiliac ligaments, and lumbar facet joints.

**Results:**

The intervention yielded substantial and sustained improvements. Pain intensity (VAS) decreased from 7 to 2, while disease activity (BASDAI) and functional impairment (BASFI) scores reduced from 3.7 to 1.3 and 4.0 to 1.0, respectively. Spinal range of motion and postural alignment were largely restored. These functional benefits proved durable over a 2-year follow-up, during which follow-up imaging confirmed persistent structural fusion, underscoring that clinical improvement was attributable to restored soft-tissue function rather than structural reversal.

**Conclusion:**

Acupotomy induced significant, long-term clinical improvement in this refractory AS case, likely through mechanical release of fascial restrictions. It represents a promising complementary, symptom- and function-modifying intervention for patients with limited response to conventional regimens.

## Introduction

Ankylosing Spondylitis (AS) is a chronic immune-mediated inflammatory disease classified under spondyloarthropathies (SpA). Its pathogenesis involves both genetic predisposition and environmental factors, such as infections (1). The human leukocyte antigen (HLA)-B27 plays a significant role in AS development. AS predominantly affects males over 30 years of age (2, 3). AS occurs more frequently in males over 30 years of age and typically originates in the sacroiliac joints, presenting as sacroiliitis and enthesitis. Common symptoms include spinal pain and morning stiffness. In advanced stages, spinal fusion may lead to kyphotic deformity, sagittal imbalance, and functional impairment (4–6).

Treatment aims to relieve symptoms, preserve spinal function, and prevent structural complications (7). Conventional management includes non-surgical and surgical approaches. Pharmacological therapies consist of nonsteroidal anti-inflammatory drugs (NSAIDs), tumor necrosis factor (TNF) inhibitors, interleukin-17 (IL-17) inhibitors, and disease-modifying antirheumatic drugs (DMARDs) for peripheral arthritis (8, 9). However, biologics are costly and not all patients respond adequately. Surgical options such as total hip arthroplasty (THA) and spinal osteotomy are reserved for severe cases but carry risks of complications (10–13). Interventional procedures including sacroiliac joint injections (SIJIs) and radiofrequency ablation (RFA) offer mainly short-term relief and thus have limited utility (14).

Complementary and Alternative Medicine (CAM) is widely adopted, particularly for pain conditions where conventional treatment proves insufficient or causes side effects (15, 16). The acupotomy technique is a form of closed release technique that integrates fundamental TCM principles with the anatomical basis of Western surgical procedures. It releases adhesions, reduces tissue pressure, downregulates inflammatory cytokines, and elevates pain thresholds. With advantages such as minimal trauma, fewer complications, and cost-effectiveness, it facilitates functional recovery in AS patients (17–20).

Guided by the “sinew-meridian theory” and a layered anatomical approach, this case report illustrates the application of acupotomy in AS, detailing localization, depth, and manipulation. Outcomes were evaluated using the Visual Analog Scale (VAS) for pain, range of motion (ROM), Bath Ankylosing Spondylitis Disease Activity Index (BASDAI), and Bath Ankylosing Spondylitis Functional Index (BASFI). This case provides a clinical reference to support the application of acupotomy therapy in the management of AS.

## Case presentation

**Figure 1:**
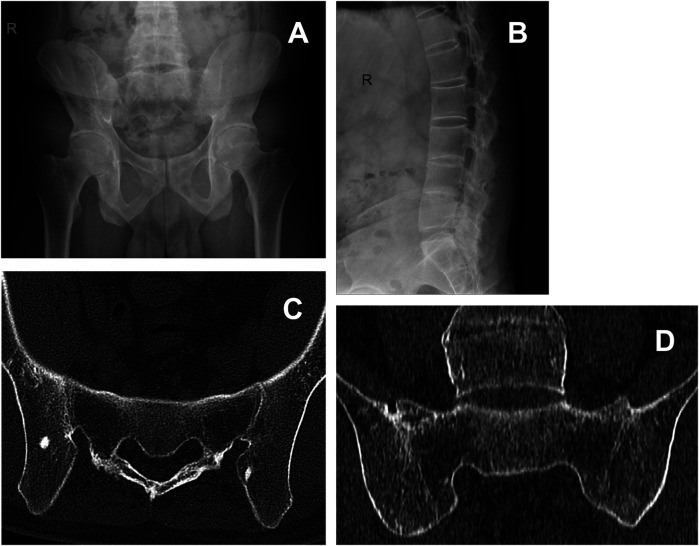
Pre-treatment radiographic assessment and two-year follow-up computed tomography (CT) in a patient with advanced ankylosing spondylitis. **(A,B)** Pre-treatment anteroposterior radiograph of the pelvis demonstrating characteristic advanced changes, including complete bony fusion of both sacroiliac joints with obliteration of the joint spaces, ankylosis of the lumbar facet joints, and a loss of the normal lumbar lordosis. **(C)** Axial CT image and **(D)** coronal CT image obtained two years post-acupotomy (2022). Both panels confirm the persistence of the bilateral sacroiliac joint bony fusion and sclerosis.

A 27-year-old male presented on June 7, 2020, with chief complaints of generalized joint pain, most prominent in the cervicothoracic and lumbosacral regions. Physical examination revealed a kyphotic posture, a barrel-shaped chest, significant cervical protraction, and an antalgic gait. He reported an inability to tolerate prolonged standing or recumbency. Radiographic examination demonstrated advanced structural involvement, including obliteration of bilateral sacroiliac joint spaces, bony fusion of the lumbar facet joints, and loss of lumbar lordosis (Figures 1A,B). No CT or MRI was performed before treatment. Therefore, while the radiographs are consistent with sacroiliac joint fusion, the exact extent of fusion and the presence or absence of active inflammatory changes could not be determined. Laboratory studies showed an elevated erythrocyte sedimentation rate (ESR) of 64 mm/h. At baseline, his symptom severity was quantified as follows: a Visual Analog Scale (VAS) score of 7, a Bath Ankylosing Spondylitis Disease Activity Index (BASDAI) score of 3.7, and a Bath Ankylosing Spondylitis Functional Index (BASFI) score of 4.0.

Before presenting to our clinic, the patient's management had been suboptimal and fragmented. He reported a history of intermittent use of non-steroidal anti-inflammatory drugs (NSAIDs), which provided only partial and transient relief of his pain and stiffness. He had also undergone several courses of traditional acupuncture therapy at local clinics, with minimal sustained benefit. Despite the radiographic evidence of advanced AS, the patient had not commenced a systematic treatment regimen with disease-modifying antirheumatic drugs (DMARDs) or biologic agents due to personal concerns regarding potential side effects and cost. It was within this context—characterized by active symptoms, significant functional impairment, and inadequate response to prior non-surgical interventions—that the patient sought acupotomy therapy as a potential minimally invasive alternative to address his debilitating pain and restricted mobility.

### Treatment

The therapeutic protocol was designed based on the principles of “meridian-specific treatment according to tendon distribution” and “anatomical layer-specific release.” The treatment sites were localized using an integrated approach grounded in meridian theory and layered anatomy. This method combined anatomical landmarking with systematic palpation to identify fascial densification along meridian pathways. Needle advancement was guided by tactile feedback from fascial resistance. Real-time imaging was not employed. Reproducibility was ensured by two key measures: using fixed surface coordinates relative to bony landmarks (e.g., the PSIS and spinous processes) and having all procedures performed by the same experienced clinician.

The treatment targeted the tendinous regions of the Foot-Taiyang, Foot-Yangming, and Hand-Sanyang. Target structures were defined by layer and region: the posterior lamina of the thoracolumbar fascia, the sacroiliac ligament complex (including the superficial and deep fibers of the posterior sacroiliac ligament and the interosseous sacroiliac ligament), focal regions of the interspinous ligaments at L4–L5/L5–S1, the facet (zygapophyseal) joint capsules, and adjacent paraspinal myofascial planes. Procedural endpoints were determined by tactile sensation: a clear fascial end-feel resistance and/or a local twitch response (LTR). The primary release sites in the sacroiliac region involved: (1) the medial attachment points of the posterior sacroiliac ligament along the inner border of the bilateral posterior superior iliac spine (PSIS; located 1.0 ± 0.3 cm medial to the PSIS); (2) the origin area of the erector spinae muscle on the dorsal sacral surface (a band-like zone 1 cm lateral to the line connecting the S1–S4 posterior sacral foramina); (3) the tender quadrants of the L4–L5/L5–S1 interspinous spaces and interspinous ligaments (within ±2 cm of the spinal intersection line connecting the highest points of the iliac crests); and (4) the transitional zone between the gluteus maximus and gluteus medius tendons at the posterior third of the iliac crest. Given the severe symptomatology and radiographic evidence of sacroiliac joint bony fusion in this case of AS, the treatment area was extended to include peripheral compensatory regions. These included the paravertebral multifidus and rotator muscles (T10–L1 segments), the cervicothoracic junction region (2 cm lateral to the C7–T3 spinous processes), the scalene–sternocleidomastoid–trapezius muscle complex (targeting the supraclavicular fossa and mastoid attachment points), and the cranial epicranial aponeurosis.

**Figure 2:**
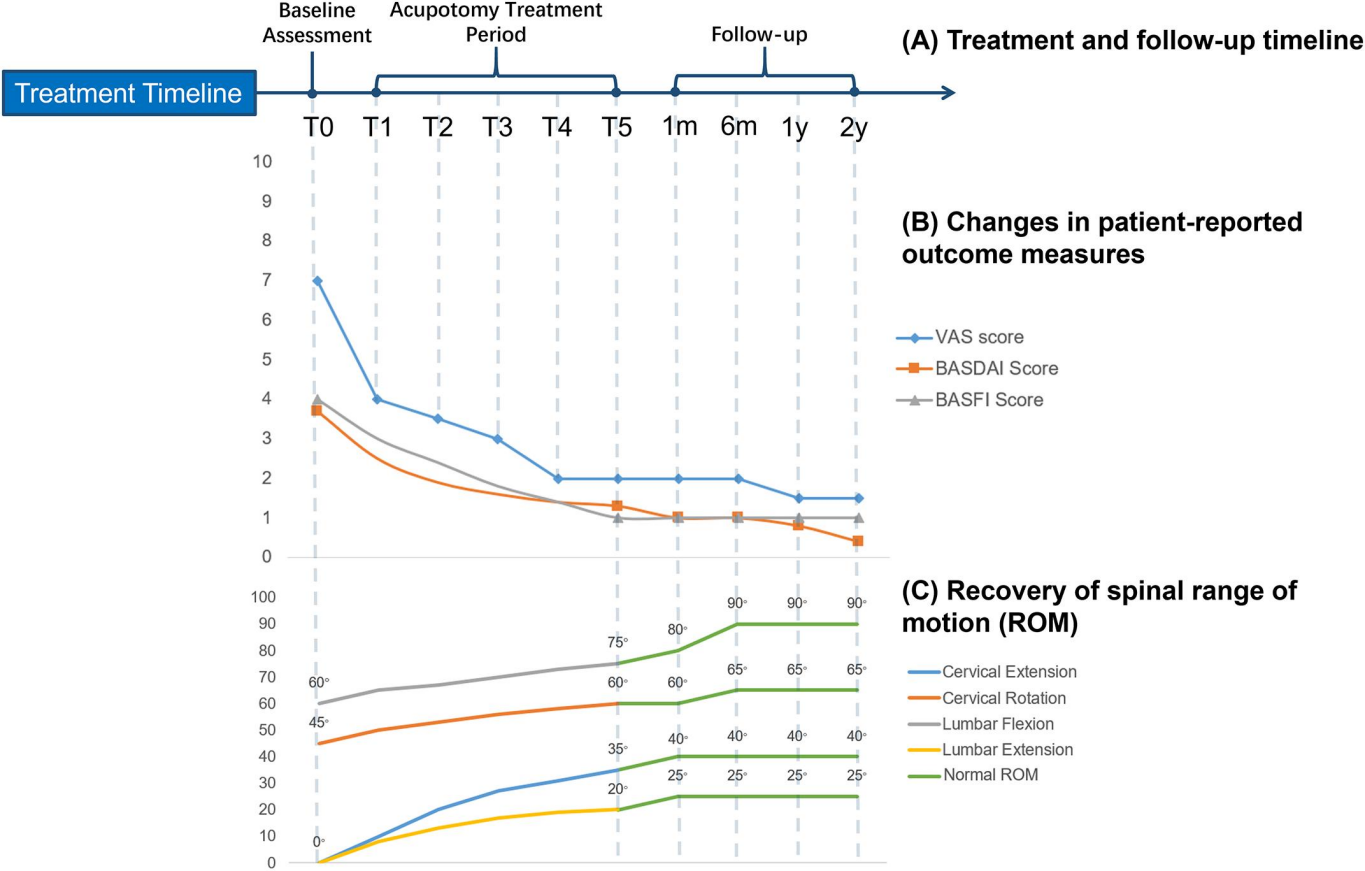
Longitudinal clinical outcomes following acupotomy therapy in a patient with advanced ankylosing spondylitis. **(A)** Schematic timeline illustrating the treatment period (five weekly acupotomy sessions, T1–T5) and follow-up assessments over two years. **(B)** Changes in patient-reported outcomes: pain intensity (visual analog scale, VAS), disease activity (bath ankylosing spondylitis disease activity index, BASDAI), and functional capacity (bath ankylosing spondylitis functional index, BASFI). A marked reduction in all scores was observed post-treatment and sustained during follow-up. **(C)** Recovery of spinal mobility. Distinct colors identify different ranges of motion (ROM) measurements. Attainment of the normal functional range is indicated by a green fill, demonstrating the progressive normalization of movement.

A systematic palpation assessment was performed before each treatment session. Constant-pressure longitudinal palpation was applied to identify tense myofascial bands, and circular palpation (with a diameter ≤2 cm) was used to detect pathological nodules. Each session targeted three to five sites with the highest comprehensive palpation scores. Treatment was administered once weekly for a total of five sessions (Figure 2A).

**Figure 3:**
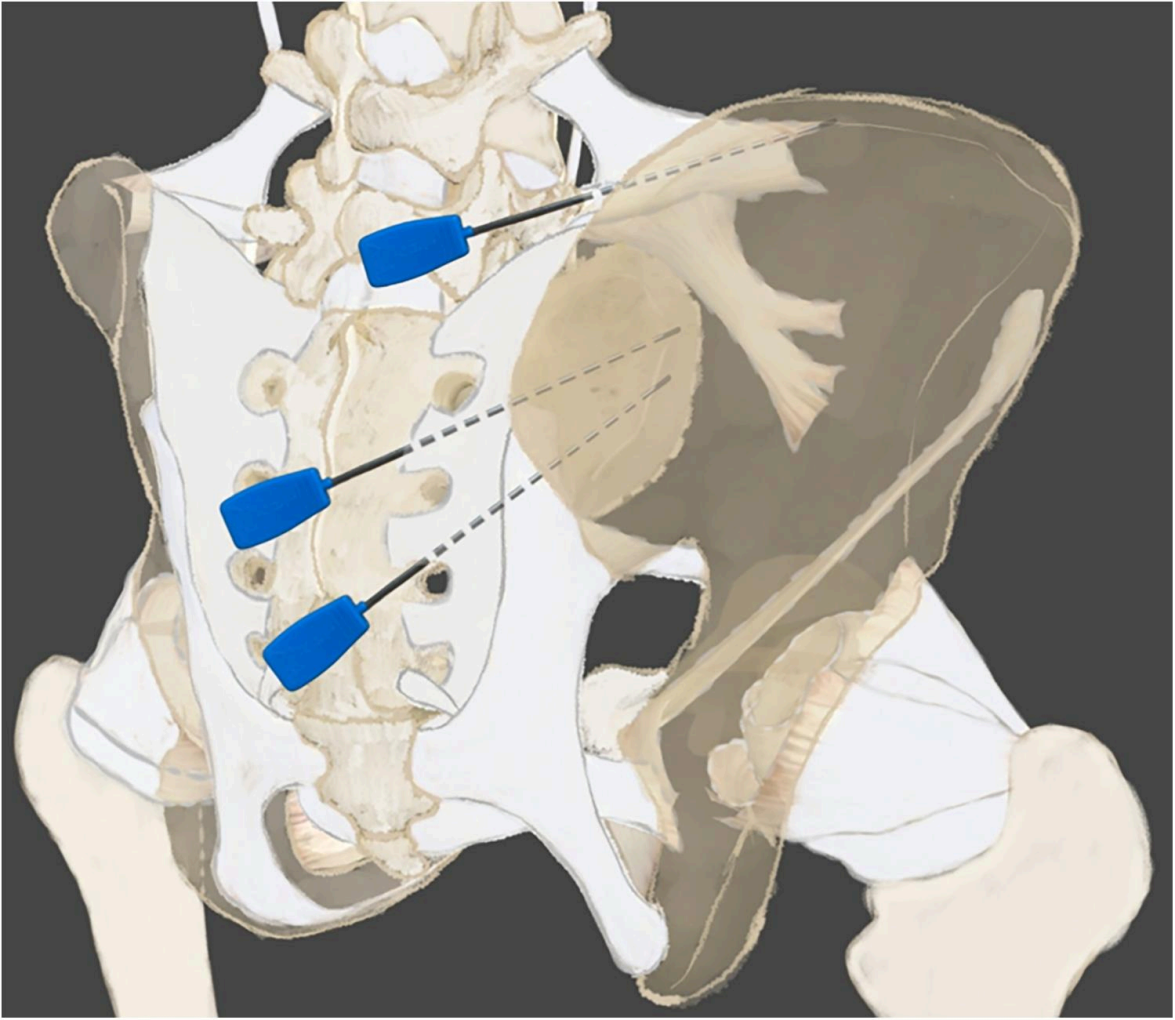
Schematic diagram of acupotomy insertion points for the sacroiliac joint. The three primary bilateral insertion points are shown in relation to key anatomical landmarks: the posterior superior iliac spine (PSIS) and posterior inferior iliac spine (PIIS). The superior, middle, and inferior points correspond to the locations for releasing the iliolumbar ligament, the posterior sacroiliac ligament near the auricular surface, and the sacroiliac ligament complex/sacrotuberous ligament, respectively. Dotted lines indicate the approximate needle trajectory and target depth.

For the ligament release procedure targeting the sacroiliac joint, six bilateral symmetric points were selected at the superior, middle, and inferior aspects (Figure 3). A 1.2 mm × 95 mm Huaxia brand acupotomy (Beijing, China) was utilized for the percutaneous release procedure. During the layered needling technique, the handle was held with a cross-grip between the thumb and index finger of the right hand, while the other fingers formed a fist. The operational procedure commenced with skin disinfection at the insertion site, with the specific needling technique varying according to the anatomical location. The superior sacroiliac point was located 1 cm superior and 0.5 cm lateral to the PSIS. The needle was inserted at a 30°–45° angle to the skin surface, with the needle tip oriented at a 30° angle to the body's longitudinal axis, allowing for rapid penetration through the skin and the overlying thoracolumbar fascia. Upon encountering a resistant barrier, the needle advancement was paused briefly for 0.1–1 s, followed by a pulsed, penetrating motion to breach the iliolumbar ligament, ultimately reaching the medial border of the ilium. A characteristic sensation of heaviness, tightness, and significant resistance to needle rotation—described as “fascial end-feel resistance”—served as the endpoint for advancement. In some instances, a local twitch response (LTR), characterized by a brief, involuntary contraction of the involved musculature, was observed visually or palpated, serving as an additional objective endpoint for adequate myofascial release. After which the needle was retained *in situ* for approximately 10 min. The middle sacroiliac point was situated 0.5–1 cm medial to the PSIS. The needle was inserted at a 45° angle to both the skin surface and the body's longitudinal axis. Needle advancement ceased upon perception of the stagnation sensation, followed by a 10-min retention period, with the depth sufficient to reach the vicinity of the auricular articular surface. The inferior point was located 1–1.5 cm medial to the posterior inferior iliac spine. Insertion was performed at a 45° angle to the skin and longitudinal axis, passing through the posterior sacroiliac ligament complex and the sacrotuberous ligament until the stagnation sensation was detected, again followed by a 10-min needle retention (Table 1).

**Table 1:** Summary of acupotomy treatment parameters, anatomical targets, and observed responses.

Treatment region/site	Anatomical landmark & insertion angle	Approximate insertion depth/layer reached	Main target structure	Instrument (diameter × length, mm)	Observed response/adverse events
Superior sacroiliac (SIJ) point	Anatomical landmark & insertion angle	Advanced to the medial border of the ilium (layer-by-layer to fascial resistance)	Iliolumbar ligament; superficial posterior sacroiliac ligament (PSL)	1.2 × 95 Huaxia acupotomy (Beijing, China)	Fascial end-feel resistance achieved; LTR elicited; no adverse events
Middle SIJ point	0.5–1 cm medial to PSIS; 45° to skin and longitudinal axis	To deep PSL/interosseous sacroiliac ligament near the auricular surface	Deep PSL; interosseous sacroiliac ligament (ISL)	1.2 × 95	Clear tissue release achieved; no adverse events
Inferior SIJ point	1–1.5 cm medial to PIIS; 45° toward the sacral base	Through the posterior SI ligament complex to the sacrotuberous ligament	Posterior sacroiliac and sacrotuberous ligaments	1.2 × 95	None
Interspinous region (L4–L5, L5–S1)	1–1.5 cm medial to PIIS; 45° toward the sacral base	To interspinous ligament and facet capsule layer	Interspinous ligament; facet (zygapophyseal) joint capsules	1.2 × 95	None
Gluteal tendon junction (posterior iliac crest)	Posterior third of the iliac crest at the junction of gluteus maximus and gluteus medius tendons	To the deep fascial plane between gluteus maximus and medius	Gluteal aponeurotic fascia and tendinous junction	1.2 × 95	None
Cranial epicranial aponeurosis	Jiao's foot motor and sensory zone: 1 cm lateral to the midline, extending 3 cm posteriorly; initial perpendicular insertion followed by sub-aponeurotic advancement	To the subaponeurotic loose connective tissue	Epicranial aponeurosis and subaponeurotic layer	1.0 × 45 Huaxia acupotomy	None

*Footnotes:*.

PIPS, posterior superior iliac spine; PIIS, posterior inferior iliac spine; SIJ, sacroiliac joint.

All procedures were performed using disposable stainless-steel acupotomy instruments (Huaxia brand, Beijing, China).

Insertion depth refers to advancement to the target fascial or ligamentous layer and may vary slightly with individual soft-tissue thickness.

No bleeding, infection, or neurological complications occurred.

Release of the cranial epicranial aponeurosis was performed at Jiao's scalp acupuncture zone, corresponding to the foot motor and sensory area. This zone is defined as extending 1 cm lateral to the midpoint of the anterior-posterior midline and continuing 3 cm posteriorly, parallel to the midline. A 1.0 mm × 45 mm Huaxia brand needle was employed. The needle was initially inserted perpendicularly through the aponeurotic layer, then leveled to a horizontal position and advanced subcutaneously within the plane between the aponeurosis and the underlying loose connective tissue. Upon sensing the characteristic stagnation, the needle was retained for 10 min and withdrawn only after a sensation of loosening was perceived at the needle tip.

Strict aseptic techniques, commensurate with standard surgical procedures, were observed throughout all interventions. Following each needling session, cupping was applied to the treated areas to extract extravasated blood. This was subsequently followed by 20 min of phototherapy using a Wirava light source directed over the treated area.

### Outcomes

Following five sessions of acupotomy therapy, the patient experienced significant alleviation of low back pain (LBP) and other AS-related systemic symptoms. Pain intensity (VAS) decreased from 7 pre-treatment to 2 post-treatment. BASFI decreased from 4.0 to 1.0 and BASDAI from 3.7 to 1.3 (Figure 2B). Clinical examination and visual estimation during follow-up demonstrated marked functional improvement: spinal ROM was largely restored to within normal limits (Figure 2C), with cervical flexion–extension improving from a severely restricted range pre-treatment to a functional range permitting overhead gaze and full chin-to-chest movement, and lumbar flexion improving from inability to reach beyond the knees to the ability to touch the toes. Postural alignment was visibly restored, including resolution of the initial kyphotic stance and cervical protraction. Although formal instrumented gait analysis, standardized endurance or balance testing were not performed, clinical observation confirmed normalization of the ambulatory pattern with resolution of an antalgic gait and restoration of symmetrical stride; the patient also reported regained capacity for prolonged standing, walking, and full return to activities of daily living and work. During the 2-year follow-up, therapeutic benefits persisted: VAS remained ≤2, BASFI and BASDAI were ≤1.0, and ROM remained within the normal range. The patient's inflammatory markers were monitored during his subsequent routine rheumatological care. While baseline C-reactive protein (CRP) level was unavailable, laboratory tests at an unspecified time point approximately two years post-acupotomy revealed an ESR of 8 mm/h and a CRP of 11.8 mg/L, indicating persistent low-grade inflammation. Subsequently, with continued standard care including regular subcutaneous injections of adalimumab, his inflammatory markers normalized, with ESR at 3 mm/h and CRP at 3.17 mg/L at a follow-up around 2025. A follow-up CT scan of the lumbosacral spine obtained two years after acupotomy (2022) confirmed the persistence of bilateral sacroiliac joint bony fusion and sclerosis, consistent with the irreversible structural damage of advanced AS. No new fractures or structural complications were identified (Figures 1C,D). These imaging findings underscore that the significant and sustained clinical improvements observed in pain, function, and mobility following acupotomy are attributable to the restoration of soft tissue mobility and biomechanical function, rather than a reversal of osseous fusion.

## Discussion

This case demonstrates that anatomy-guided acupotomy can induce significant and sustained improvements in pain, disease activity, and spinal mobility in a patient with advanced, treatment-refractory AS. The stability of these benefits over a 2-year follow-up suggests a durable therapeutic effect beyond transient symptom modulation.

From the perspective of modern fascial science, the therapeutic mechanism of acupotomy is considered to involve the mechanical alteration of pathologically densified fascia. In ankylosing spondylitis, enthesitis-driven fibrosis produces a state of fascial densification, evidenced by increased thickness, stiffness, and impaired inter-layer gliding (21). Acupotomy is directed at these dysfunctional areas within biomechanically pivotal structures like the thoracolumbar fascia and sacroiliac ligaments (22). The rationale for this approach is supported by findings that such fibrosis directly compromises tissue gliding and promotes stiffness. The restoration of mechanical mobility through acupotomy is thus proposed to contribute to the normalization of aberrant mechanoreceptor signaling within the fascia, offering a plausible explanation for the observed alleviation of pain, stiffness, and impaired proprioception (23).

From a biomechanical perspective, acupotomy is theorized to mitigate the pathological stiffness characteristic of Ankylosing Spondylitis (AS) by targeting the altered connective tissue structure. The intervention is hypothesized to achieve mechanical release of contracture nodules and restore inter-layer gliding through its precise cutting action. Chronic inflammation in AS leads to fibrosis within the fascial system, particularly in the thoracolumbar fascia (TLF), which is integral for load transfer. The sacroiliac joint (SIJ) acts as a biomechanical pillar for posture maintenance, compensating for lost spinal mobility post-fusion and serving as a cornerstone for lumbopelvic stability (24). Langevin et al. demonstrated structural pathology in the lumbar fascial system, with increased thickness and echogenicity of perimuscular connective tissue indicative of tissue disorganization and fibrosis that can impair this SIJ-centric stability system (21). Acupotomy, by performing precise mechanical interventions in the TLF, aims to restore tissue mobility and optimize force transmission through this SIJ pillar. This release may extend its influence to the facet joint chain, a critical determinant of spinal segmental motion and load distribution, by reducing asymmetric stresses on their capsules and articular surfaces, thereby promoting more harmonious spinal alignment and movement (25). Release of posterior TLF and PSL/ISL adhesions likely restored layer sliding and reduced facet-capsular strain at L4–S1, which may underlie the clinical improvement. The functional gains in posture and mobility, observed alongside radiologically persistent fusion, strongly suggest that acupotomy's efficacy stems from restoring pliable function to the investing fascial system, rather than reversing the established bony ankylosis.

Concurrently, acupotomy operates through neurophysiological pathways. The altered connective tissue environment in AS creates fertile ground for peripheral and central sensitization. The procedure is further hypothesized to achieve decompression of nociceptors while modulating proprioceptive and autonomic tone within the fascial system. The fascia investing the biomechanically pivotal SIJ and the richly innervated facet joint capsules in pathological states can lead to persistent nociceptive input (21, 24). The mechanical stimulation from acupotomy disrupts this pain cycle. While distinct from dry needling, the therapeutic principle of precise mechanical disruption of myofascial dysfunction is conceptually parallel. In this context, studies on ultrasound-guided dry needling highlight the critical importance of accuracy, demonstrating that targeted intervention eliciting local twitch responses correlates strongly with superior pain relief (26). In this case, the occasional elicitation of LTR during acupotomy provided an objective sign of adequate myofascial release, correlating with the subsequent clinical improvement. This underscores the broader neurophysiological principle that precise biomechanical intervention can effectively reduce sustained nociceptive barrage. Furthermore, the controlled microtrauma induced by acupotomy may stimulate a localized healing response, modulating the inflammatory milieu and promoting the release of endogenous opioids, leading to desensitization of peripheral nociceptors and alleviating pain from the biomechanically stressed SIJ and facet joint environment (27).

Compared to Ultrasound-Guided Dry Needling (DN-US) and Ultrasound Neuromodulation Knife Therapy (US-NKT), acupotomy is more invasive as it involves mechanical release of fascial adhesions, particularly in structures like the thoracolumbar fascia (TLF), to restore tissue mobility and optimize force transmission through key biomechanical pillars such as the sacroiliac joint (SIJ). While DN-US primarily targets myofascial trigger points (MTrPs) under ultrasound guidance to elicit local twitch responses (LTRs) and relieve pain, it does not address the structural fibrosis and adhesions characteristic of chronic conditions like Ankylosing Spondylitis (AS). Ultrasound guidance significantly enhances accuracy and reduces procedural risks by allowing real-time visualization of anatomical structures and pathological changes, as demonstrated by the superior outcomes in pain reduction and LTR elicitation with DN-US compared to non-guided methods. However, DN-US was not adopted in this context because its mechanism is limited to neuromuscular modulation and MTrP inactivation, without the biomechanical capacity to release deep fascial restrictions. In contrast, acupotomy's precise mechanical intervention targets both neurophysiological sensitization and biomechanical dysfunction, making it more suitable for addressing the fibrotic and stiffened connective tissue in AS (26, 28).

From a rheumatologist's perspective, integrating therapeutic drug monitoring (TDM) of biopharmaceuticals with acupotomy offers a stratified approach to managing ankylosing spondylitis, particularly in patients with persistent inflammation, suboptimal biologic response, or structural fusion. TDM guides pharmacologic optimization, while acupotomy provides a minimally invasive means to release fibrotic tension in key structures such as the thoracolumbar fascia and sacroiliac joint—potentially delaying or supplementing surgical intervention by restoring tissue mobility and mitigating nociceptive drive. When combined with postural retraining and neuromuscular re-education, this multimodal strategy supports functional recovery and long-term stability, aligning with a personalized, mechanism-based treatment paradigm (29–31).

It is important to note that the acupotomy procedures in this case were performed based on meticulous palpation and anatomical landmarks, without real-time ultrasound guidance. This approach reflects the common clinical practice of acupotomy in many settings and relies on the practitioner's kinesthetic feedback to navigate fascial layers. While this method proved effective in our case, the adoption of ultrasound guidance in future studies could serve two critical purposes. First, to objectively confirm the precise placement of the acupotomy needle within the targeted pathological fascia or ligament, thereby enhancing procedural accuracy and safety. And second, to provide direct visual evidence of the immediate biomechanical changes, such as fascial release and tissue gliding, thus offering tangible proof for the proposed mechanism of action. Future work will integrate functional ultrasound (fUS) to visualize fascial layer motion during acupotomy.

## Conclusion

This case demonstrates that acupotomy therapy, guided by meridian tendon theory and anatomical release principles, can produce significant and sustained symptomatic and functional improvements in advanced AS, even in the presence of irreversible structural damage. It represents a potent symptom- and function-modifying intervention that complements, rather than replaces, pharmacologic control of inflammation. Its role is particularly valuable for patients with contraindications or inadequate response to conventional treatments, offering a mechanical solution to the functional limitations that often persist despite biologic therapy.

### Limitations

This study has several limitations. The absence of real-time ultrasound or MRI for fascial assessment means we could not objectively visualize the immediate biomechanical release or quantify baseline fascial pathology. Furthermore, local twitch responses (LTRs) were not systematically recorded, lacking a key objective endpoint for myofascial release. The follow-up of objective inflammatory markers was also incomplete, limiting our ability to correlate clinical improvement with changes in systemic inflammation. Future research should employ multimodal imaging, such as sonoelastography and fascial thickness mapping, to objectively quantify tissue properties. Ultimately, large-scale randomized controlled trials (RCTs) that integrate these imaging techniques with biomechanical and inflammatory parameters are essential to validate the efficacy and mechanisms of acupotomy in AS.

## Data Availability

The datasets generated and analyzed during this case report are not publicly available due to patient privacy concerns. De-identified data may be made available from the corresponding author upon reasonable request and with permission of the patient.
